# Fusing Metals in Tin

**Published:** 1900-02-15

**Authors:** 


### FUSING METALS IN TIN.

But one experiment which was made in connection with this writing may astonish others as it did myself. It was a test case, not for compounding in proportions for alloy, but to determine the time of fusing platinum, gold, silver and copper in tin. A small sand crucible was used which would hold about two ounces of metal. About one ounce of tin was used, and strips of metal platinum, gold, silver and copper prepared two inches long, one-eighth inch wide and No. 34 plate gauge in thickness, except the platinum, which was rolled to 36. The crucible contained borax to about one-third full when fused. The tin was dropped in, which was covered with the molten salts. Repetitions were made to time the disappearance of the other metals. The platinum was first placed upright, and it extended above the flux nearly one inch and a quarter. In ten seconds it was out of sight. The gold, silver and copper strips were introduced in like manner, and all were taken down in a second's time. In practical work sixty-eight dwt. pure silver of 37 gauge, when cut into strips or loosely rolled, will be fused in twenty-five dwts. of tin in from five to ten minutes.

It is a fact that the above named metals may be compounded with no higher heat than is required to maintain the fluidity of borax. Any one can easily become convinced by trying the experiment.—*S. B. Palmer, in Items of Interest.*

